# Solitary, subcutaneous, fixed, firm, and fast-growing nodule

**DOI:** 10.1016/j.jdcr.2022.02.040

**Published:** 2022-03-26

**Authors:** Moatasem Hussein Al-Janabi, Noura Ali, Oudae Mohammad Yousof, Zuheir Al-Shehabi, Fouz Hasan

**Affiliations:** a Department of Pathology, Cancer Research Center, Tishreen University Hospital, Lattakia, Syria; b Department of Dermatology, Cancer Research Center, Tishreen University Hospital, Lattakia, Syria; c Department of Plastic Surgery, Tishreen University Hospital, Lattakia, Syria; d Department of Dermatology, Tishreen University Hospital, Lattakia, Syria

**Keywords:** basaloid cells, necrosis, nodules, subcutaneous

## Case

**Fig 1:**
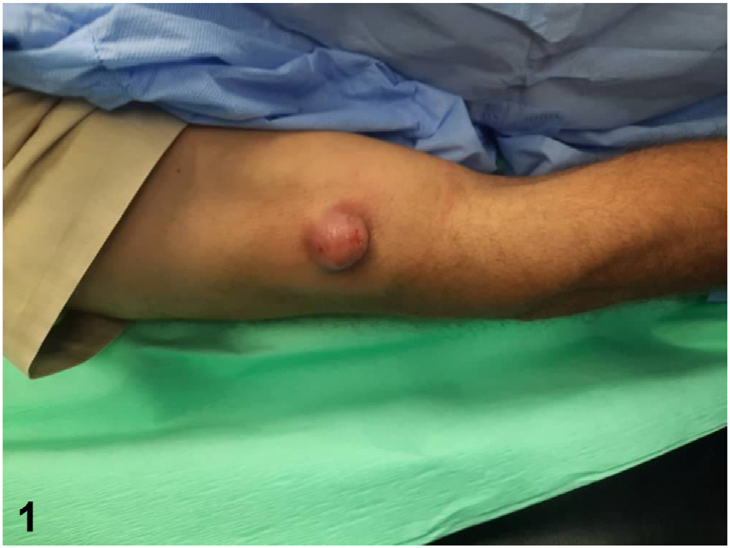

**Fig 2:**
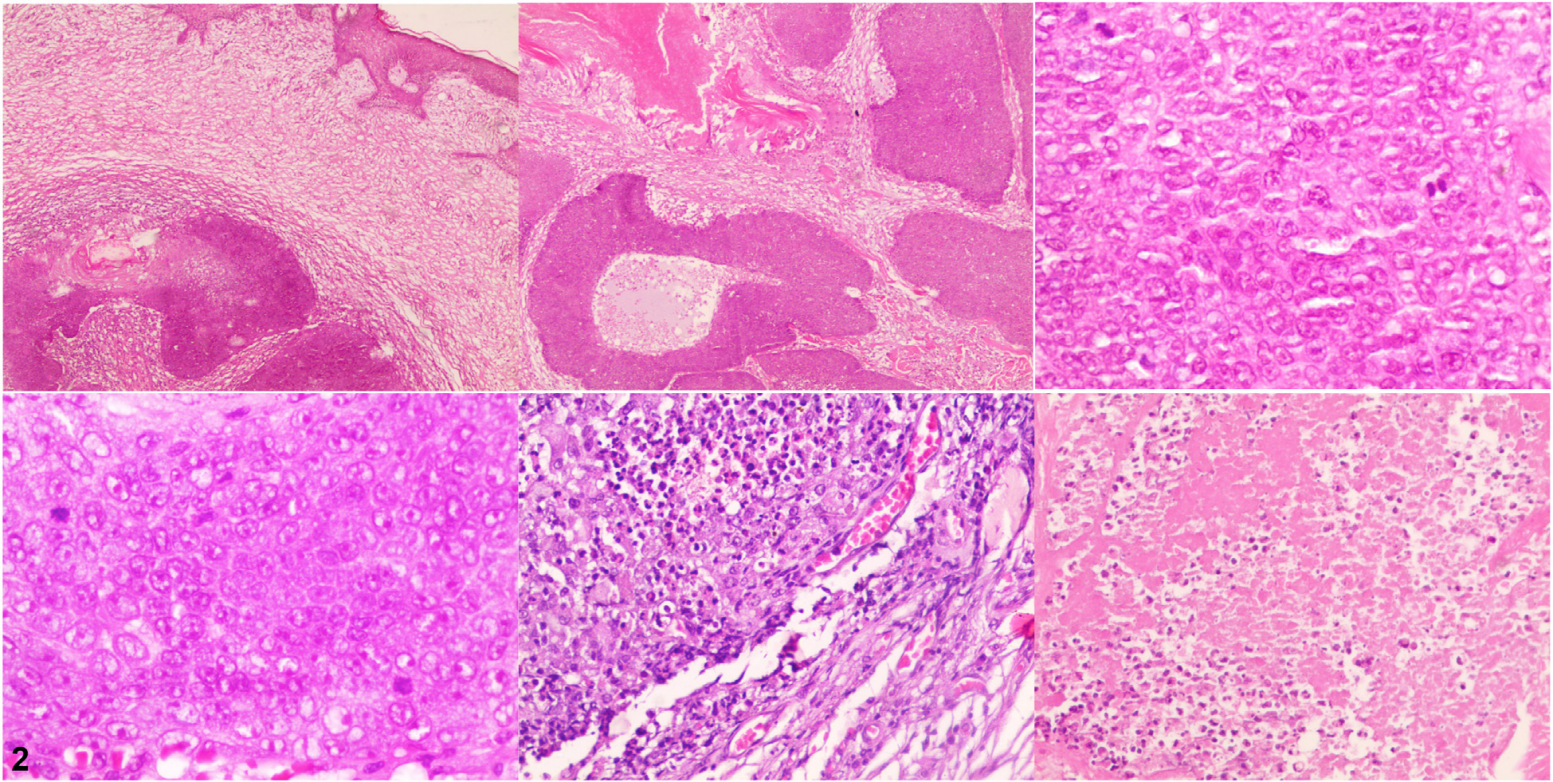

**Fig 3:**
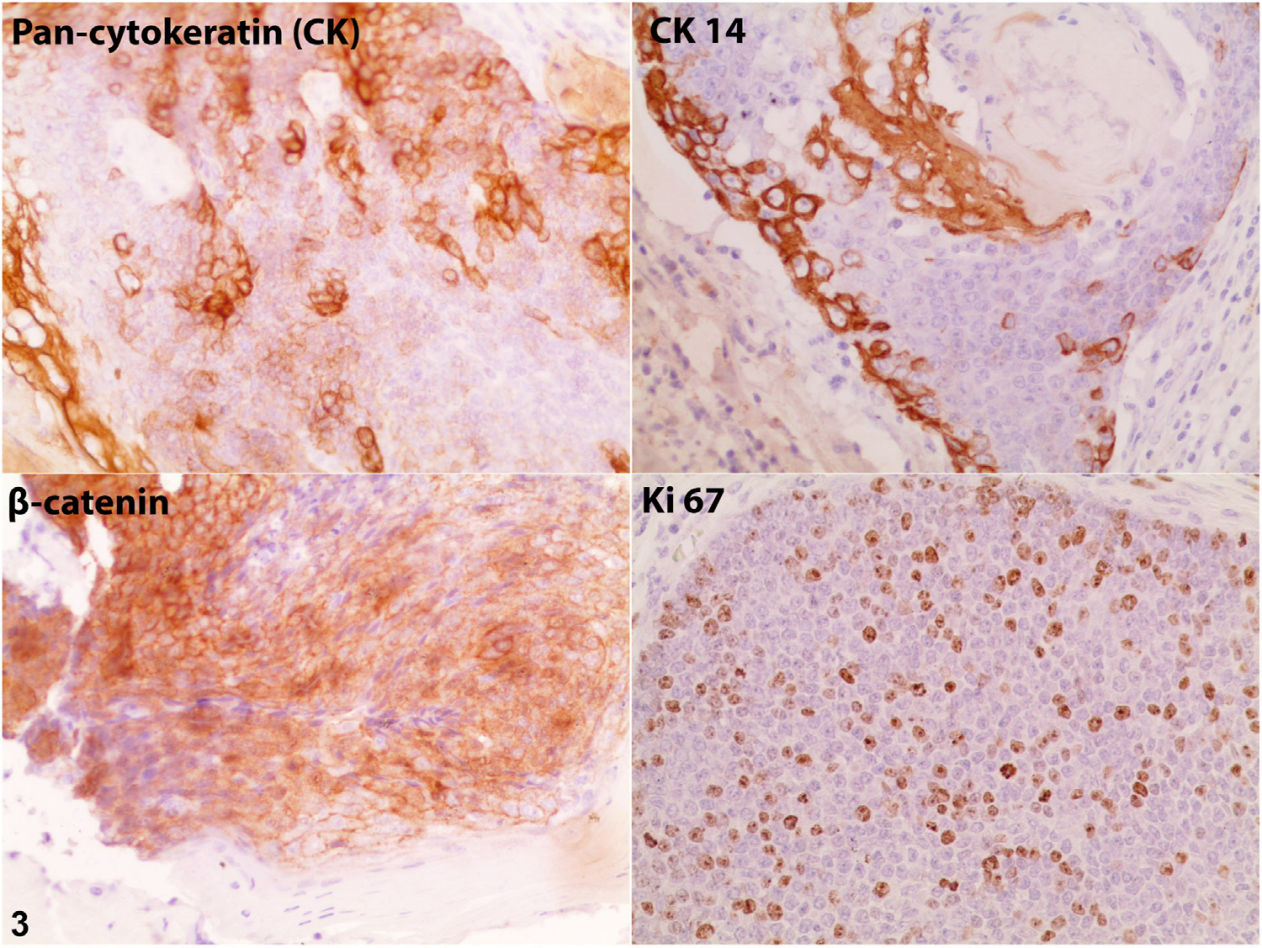

A 59-year-old man with hypertension and diabetes mellitus type 2 presented to the dermatology clinic with a solitary, subcutaneous, asymptomatic, fast-growing nodule on the lower part of his right arm, near the elbow. The nodule had been present for 2 months. A clinical examination revealed that the nodule was red, fixed, firm, well defined, and approximately 5.0 cm in diameter (Fig 1). A routine blood investigation (complete blood cell count) revealed no abnormalities. An excisional biopsy was obtained, and hematoxylin-eosin and immunohistochemical staining were performed (Fig 2 [200×] and Fig 3).

**Question 1: What is the most likely diagnosis?**

A. Basal cell carcinoma
B. Proliferating pilomatrixoma (aggressive type)
C. Malignant pilomatrixoma
D. Trichoepithelioma
E. Trichilemmal carcinoma

**Answers:**

A. Basal cell carcinoma – Incorrect. Basal cell carcinoma is characterized by basal cell proliferation in continuity with the surface epidermis, peripheral nuclear palisading, and retraction spaces between the epithelium and the stroma.^1^
B. Proliferating pilomatrixoma (aggressive type) – Correct. Proliferating pilomatrixoma is an uncommon, benign variant of pilomatrixoma with atypical features, including basaloid cell pleomorphism, loss of polarity, nuclear hyperchromatism, infiltration of the dermal collagen, extensive necrosis (Fig 2), and a high mitotic index (Ki-67 in Fig 3).^2^
C. Malignant pilomatrixoma – Incorrect. Malignant pilomatrixoma is a very rare malignant neoplasm arising from the hair matrix. It is composed of solid nests of basaloid cells with marked nuclear pleomorphism, prominent nucleoli, abnormal frequent mitoses, involvement of the fascia or skeletal muscle, stromal desmoplasia, and vascular lymphatic or perineural invasion. Tumor lobules are irregular. These histologic findings were not seen in this case.^3^
D. Trichoepithelioma – Incorrect. Trichoepithelioma is a benign neoplasm with follicular differentiation, composed of horn cysts and islands of basaloid cells surrounded by a fibroblastic stroma. The keratinization is abrupt and complete, and shadow cells are not seen.^4^
E. Trichilemmal carcinoma – Incorrect. Trichilemmal carcinoma is an uncommon, malignant, cutaneous, adnexal tumor derived from the external root sheath of the hair follicle, mainly found on the sun-exposed skin of the elderly. It measures 0.5 to 2.0 cm. Microscopically, tumor lobules are characterized by a peripheral palisade of cuboidal or columnar cells with foci of clear cells, and the polarity of peripheral cells is reversed. Basaloid cell predominance may be seen.^4^

**Question 2: Which of the following histopathologic findings is characteristic of the tumor?**

A. Nuclear palisading
B. Keratin pearls
C. Ghost “shadow” cells
D. Horn cysts
E. Pushing and rolling border containing a clear cell population

**Answers:**

A. Nuclear palisading – Incorrect. Nuclear palisading is seen in basal cell carcinoma.
B. Keratin pearls – Incorrect. Keratin pearls are found in squamous cell carcinoma.
C. Ghost “shadow” cells – Correct. Ghost or shadow cells are characteristic of pilomatrixoma.
D. Horn cysts – Incorrect. Horn cysts are seen in trichoepithelioma.
E. Pushing and rolling border containing a clear cell population– Incorrect. This feature belongs to trichilemmal carcinoma.

**Question 3: Which of the following is considered the gold standard for treating this tumor?**

A. Narrow-margin excision
B. Wide local excision with confirmed negative margins
C. Radiotherapy
D. Chemotherapy
E. Imatinib

**Answers:**

A. Narrow-margin excision – Incorrect. Local recurrence is common in proliferating pilomatrixoma; therefore, excision with narrow margins is not recommended.^5^
B. Wide local excision with confirmed negative margins – Correct. Local recurrence is common in proliferating pilomatrixoma; therefore, wide local excision with confirmed negative margins is the treatment of choice for this tumor.^5^
C. Radiotherapy – Incorrect. Usually, no adjuvant therapy is necessary.^5^
D. Chemotherapy – Incorrect. Usually, no adjuvant therapy is necessary.^5^
E. Imatinib – Incorrect. Imatinib is an oral tyrosine kinase inhibitor. It has received approval for the treatment of several oncologic conditions. It is indicated for adult dermatofibrosarcoma protuberans.

## Conflicts of interest

None disclosed.
